# Evidence for Paracrine Protective Role of Exogenous αA-Crystallin in Retinal Ganglion Cells

**DOI:** 10.1523/ENEURO.0045-22.2022

**Published:** 2022-03-04

**Authors:** Madhu Nath, Zachary B. Sluzala, Ashutosh S. Phadte, Yang Shan, Angela M. Myers, Patrice E. Fort

**Affiliations:** 1 Department of Ophthalmology and Visual Sciences, University of Michigan, Ann Arbor, MI 48105; 2 Currently in Department of Biochemistry and Molecular Biology, Thomas Jefferson University, Philadelphia, PA 19107; 3 Department of Molecular and Integrative Physiology, University of Michigan, Ann Arbor, MI 48105

**Keywords:** αA-crystallin, chaperone, metabolic stress, neuroprotection, recombinant proteins

## Abstract

Expression and secretion of neurotrophic factors have long been known as a key mechanism of neuroglial interaction in the central nervous system. In addition, several other intrinsic neuroprotective pathways have been described, including those involving small heat shock proteins such as α-crystallins. While initially considered as a purely intracellular mechanism, both αA-crystallins and αB-crystallins have been recently reported to be secreted by glial cells. While an anti-apoptotic effect of such secreted αA-crystallin has been suggested, its regulation and protective potential remain unclear. We recently identified residue threonine 148 (T148) and its phosphorylation as a critical regulator of αA-crystallin intrinsic neuroprotective function. In the current study, we explored how mutation of this residue affected αA-crystallin chaperone function, secretion, and paracrine protective function using primary glial and neuronal cells. After demonstrating the paracrine protective effect of αA-crystallins secreted by primary Müller glial cells (MGCs), we purified and characterized recombinant αA-crystallin proteins mutated on the T148 regulatory residue. Characterization of the biochemical properties of these mutants revealed an increased chaperone activity of the phosphomimetic T148D mutant. Consistent with this observation, we also show that exogeneous supplementation of the phosphomimetic T148D mutant protein protected primary retinal neurons from metabolic stress despite similar cellular uptake. In contrast, the nonphosphorylatable mutant was completely ineffective. Altogether, our study demonstrates the paracrine role of αA-crystallin in the central nervous system as well as the therapeutic potential of functionally enhanced αA-crystallin recombinant proteins to prevent metabolic-stress induced neurodegeneration.

## Significance Statement

αA-crystallin is a chaperone protein that has been long known for its critical role in the lens proteostasis. Recent studies have highlighted the protective potential of αA-crystallin in the central nervous system, especially the retina. The broad chaperone and cytoprotective functions of αA-crystallin make it a very attractive target in the context of the dire need for novel protective therapies for neurodegenerative diseases. Our previous work has shown that phosphorylation on threonine 148 (T148) is a critical regulator of the cytoprotective function of αA-crystallin. The current study demonstrates that αA-crystallin secreted by Müller glial cells (MGCs) plays a paracrine protective role for retinal neurons. We further demonstrated the therapeutic potential of a functionally enhanced αA-crystallin recombinant protein in promoting neuronal survival.

## Introduction

α-Crystallins (αA- and αB-) have been extensively described as resident chaperone proteins in the eye lens and are imperative for maintaining transparency (Masilamoni et al., 2005; Hejtmancik et al., 2015; Makley et al., 2015; Ghosh and Chauhan, 2019). In recent years, both proteins gained substantial interest in the context of retinal insults and neurodegenerative diseases (Ying et al., 2008; Munemasa et al., 2009; Wang et al., 2011; Piri et al., 2013; Zhu and Reiser, 2018). Although the presence of α-crystallins was initially described and studied in the ocular lens, their expression is not limited to this tissue. αB-crystallin is ubiquitously expressed or stress-induced in most tissues and cells, including heart, skeletal muscle, kidney, lung, brain, and retina. αA-crystallin, however, is basally expressed at low levels in a limited number of tissues while highly induced under stress conditions in the kidney and the central nervous system, including the retina (Dubin et al., 1991; Zhang et al., 2019). In the retina, both α-crystallin proteins have been found predominantly in glia and retinal ganglion cells (RGCs) in the inner retina, as well as photoreceptors and retinal pigmental epithelium (RPE) in the outer retina (Rao et al., 2008; Munemasa et al., 2009; Kase et al., 2012; Shi et al., 2015; Kannan et al., 2016; Ruebsam et al., 2018). Initially thought to be products of gene duplication, both αA-crystallins and αB-crystallins are now known to present different expression patterns and functional roles, independent from each other, including in neuroprotection (Robinson and Overbeek, 1996).

The neuroprotective function has been recently linked to both αA-crystallins and αB-crystallins in the context of different neurodegenerative diseases (Kannan et al., 2016; Zhu and Reiser, 2018). Proposed mechanisms for these neuroprotective functions of α-crystallin proteins include attenuation of mitochondrial dysfunction (Zhu and Reiser, 2018), reduced accumulation of misfolded proteins (Schmidt et al., 2012), and specific disruption of neuronal apoptotic pathways (Piri et al., 2013, 2016; Hua Wang et al., 2020). Additionally, studies have established a strong relationship between these two protein’s expression and chaperone activity and their observed anti-apoptotic function (Pasupuleti et al., 2010; Piri et al., 2013). As members of the small heat shock protein family, α-crystallins have been shown to prevent protein aggregation as well as promote cell survival under conditions such as chemically induced hypoxia (Yaung et al., 2008; Schmidt et al., 2012) including through inhibition of apoptosis. Expression of αA-crystallins and αB-crystallins have been shown to increase in an experimental model of light-induced damage to the retina (Heinig et al., 2020), as well as at different stages of the wound healing process following retinal tear (Baba et al., 2015). Consistent with a protective potential for retinal neurons, α-crystallin expression was also shown to correlate with increased RGC survival following optic nerve axotomy (Munemasa et al., 2009) and in rescuing photoreceptors in a light-induced damage model (Heinig et al., 2020).

Studies from our lab and others have reported an increased αA-crystallin expression in the retinas of diabetic rodents as well as human donors with diabetes (Fort et al., 2009; Ruebsam et al., 2018). However, αA-crystallin function seemed to be impaired in the diabetic retina, as suggested by loss of solubility, and change in their posttranslational modification (PTM) pattern (Reddy et al., 2013). PTMs have been reported to not only influence the structure but also the neuroprotective and chaperone functions of α-crystallins (Kim et al., 2007; Heise et al., 2013). Specifically, phosphorylation on serine residues 19, 45, and 59 of αB-crystallin (Kim et al., 2007; Heise et al., 2013; Reddy et al., 2013) and residues 122 and 148 of αA-crystallin seem to be critical regulators of their chaperone and protective functions (Ruebsam et al., 2018). Interestingly, while previous studies have shown increased phosphorylation for αB-crystallin (Heise et al., 2013; Reddy et al., 2013), αA-crystallin phosphorylation on residue 148 was dramatically reduced in the retina from diabetic rodents and diabetic donors, especially those with retinopathy (Ruebsam et al., 2018). We also showed that the threonine 148 (T148)D phosphomimetic form of αA-crystallin is a potent neuroprotector for retinal neurons against serum deprivation-induced cell death (Ruebsam et al., 2018). Furthermore, we have demonstrated that glial cells overexpressing αA-crystallin secrete the protein in their extracellular environment and that supplementation of conditioned media from these cells efficiently promotes R28 cell survival following exposure to serum starvation-induced apoptotic stress (Ruebsam et al., 2018). Interestingly, these observed anti-apoptotic effects were only observed from cells expressing the wild-type (WT) or phosphomimetic (T148D) protein, but not its nonphosphorylatable counterpart (T148A). While this pointed to a critical role of this phosphorylation, the impact of this posttranslational modification on the structure-function relationship of αA-crystallin remains unknown.

In all, our current understanding of α-crystallin function draws out two major observations that (1) αA-crystallin serves a key neuroprotective function within the retinal tissue and (2) controlling/enhancing αA-crystallin function presents the high potential to promote retinal cell survival and maintenance of the microarchitecture of the neuroretina. Therefore, in the present study, we studied the impact of T148D mutation of αA-crystallin on its chaperone function and associated alteration of its biochemical properties. Furthermore, we assessed the potential of supplementation with recombinant T148D αA-crystallin protein to promote survival of retinal neurons, especially primary RGCs, following exposure to metabolic stress. The current study, therefore, unveils an exciting new avenue for the use of αA-crystallin and its functionally enhanced derivatives to slow the progression of retinal neurodegenerative disorders.

## Materials and Methods

### Cell lines

Rat retinal Müller cells (rMC-1) and retinal neuronal cell (R28) lines were obtained from Applied Biological Materials Inc. All cell lines were maintained in DMEM, 5 mm glucose (DMEM-NG) supplemented with 10% fetal bovine serum (FBS; Flow Laboratories) at 37°C, 5% CO_2_ unless stated otherwise. For experiments, R28 cells were differentiated into neurons in DMEM with 8-(4-chlorophenylthio) cAMP (8-CPT-cAMP; catalog #C3912, Millipore Sigma) at a final concentration of 2.5 mm on laminin-coated plates as described earlier (Ruebsam et al., 2018).

Primary Müller glial cells (MGCs) were obtained from the αA-crystallin knock-out (AKO) mice originally generously provided by Wawrousek from the National Eye Institute (NEI). Cells were isolated using a protocol adapted from Hicks and Courtois (Hicks and Courtois, 1990) and characterized previously (Brady et al., 1997). Briefly, primary MGCs were isolated from the retinal tissue of Post-natal day (P10–P14) AKO mice pups and maintained in DMEM-NG + 10% FBS + 1% penicillin/streptomycin (catalog #15140122, Thermo Fisher Scientific). The purity and specificity of the cell preparation were validated by evaluating the expression of the Müller cell-specific markers glutamine synthetase, Prdx-6, and Abc8a from Passage 2–6 as described previously (Nath et al., 2021).

### Primary RGCs

RGCs were isolated and purified from AKO mice pups at P3–P5 using a modified immunopanning method described previously (Winzeler and Wang, 2013). The purified RGCs were resuspended in growth media containing B27 supplement (Thermo Fisher Scientific), 50 ng/ml BDNF (catalog #B3795, Millipore Sigma), 10 ng/ml CNTF (catalog #C3835, Millipore Sigma), and 4 μg/ml forskolin (catalog #F3917, Millipore Sigma) before being seeded onto poly-D-lysine and laminin-coated glass coverslips in 24-well culture plates. Cells were seeded at a density of 30,000 cells/cm^2,^ and the growth media was changed every 3 d until use.

### Transient transfection of rMC-1 and MGCs and recovery of conditioned media

Cells were transfected using the Neon Transfection System (Invitrogen) following the manufacturer’s instructions. Briefly, cells were trypsinized and washed in PBS before being resuspended in 110-μl resuspension buffer and electroporated with 2.5 μg of the previously characterized pcDNA 3.1 vectors expressing either WT, the phosphomimetic T148D, or the nonphosphorylatable T148A crystallins, respectively (Ruebsam et al., 2018) and were seeded in six-well plates. The next day, transfected cells were incubated either in serum-free DMEM-NG (with 20 mm mannitol), DMEM-HG (DMEM-NG with 20 mm glucose), or DMEM-HG with 100 ng/ml TNFα (catalog #210-TA, R&D Systems) for 24 h. Cells incubated in DMEM-NG served as the experimental control.

Following incubation, growth media from transfected glial cells was recovered and prepared as previously described (Ruebsam et al., 2018). Briefly, the media was first filtered using a 0.22-μm syringe filter (catalog SLMP025SS, Millipore Sigma) and then centrifuged sequentially at 300 × *g* for 6 min, 3000 × *g* for 20 min, and 5000 × *g* for 10 min at room temperature. Finally, the media were concentrated using 3K MWCO concentrators (catalog #C775, Amicon, Merck Millipore) and stored at 4°C until use in conditioned media experiments.

### Generation of recombinant αA-crystallins

pET23d+ vectors containing the cDNA sequence for human αA-crystallin were used as a template for generating mutant proteins. Point mutations on T148 corresponding to the phosphomimetic (T148D) and the nonphosphorylatable (T148A) analog of αA-crystallin were introduced using the QuikChange Site-directed mutagenesis kit (Agilent Technologies) using primers listed in Table 1. Cloned plasmids were scaled up in XL-1 Blue supercompetent cells, and isolated plasmid sequences were confirmed by Sanger sequencing. Sequenced plasmids were then used to transform BL21(DE3) pLysS cells to optimize the respective proteins’ expression.

**Table 1:** Primers used for point mutations on T148 corresponding to the phosphomimetic (T148D) and the nonphosphorylatable (T148A) analog of αA-crystallin

Protein	Primers
T148A	5'-gcatccaggccagcctggatcttgggg-3'
	5'-ccccaagatccaggctggcctggatgc-3'
T148D	5'-gtggcatccaggccatcctggatcttggggcc-3'
	5'-ggccccaagatccaggatggcctggatgccac-3'

Cells were grown in LB Miller broth (catalog #BP142610P1, Fisher Scientific) in a rotary shaker maintained at 37°C, 225 rpm, till they reached an OD_600_ between 0.4 and 0.6. Protein expression was induced by the addition of isopropyl-β-D-thioglalctopyranoside (IPTG; catalog #I2481C, GoldBio) at a final concentration of 500 μm for 4 h. Bacterial cell pellets were harvested by centrifugation at 4000 rpm for 20 min at 4°C and stored overnight at –80°C. Cell lysis and protein purification were conducted using size exclusion chromatography as described previously (Horwitz et al., 1999). Purified proteins were subjected to endotoxin removal using Triton X-114-mediated phase separation using a protocol adapted from Teodorowicz et al. (2017). The efficacy of endotoxin removal was ascertained using the Pierce Chromogenic Endotoxin Quant kit (catalog #A39552, Thermo Fisher Scientific) as per manufacturer’s instructions. Protein purity was assessed by Coomassie Blue staining and Immunoblot analysis (Fig. 3*A*). All proteins were stored in PBS pH 7.4 at –80°C until use.

### Chaperone activity assay

The functional efficacy of purified αA-crystallins to prevent nonspecific protein aggregation *in vitro* was assessed by chaperone assays as described previously (Horwitz, 1992). Aggregation of 75-μg alcohol dehydrogenase (ADH) in PBS pH 7.4 against varied amounts of αA-crystallin was chemically induced by adding EDTA at a concentration of 37.5 mm. Protein aggregation was monitored as relative absorbance at 360 nm in a FLUOstar OMEGA plate reader (BMG Labtech). Representative assays are an average of three independent experiments for statistical significance.

### Native gel electrophoresis

The polydispersity profile of purified αA-crystallins *in vitro* was assessed by Native PAGE gels. Samples were prepared using 7.5 μg of recombinant WT, T148D, or T148A αA-crystallin resuspended in Novex Tris-Glycine Native Sample buffer (2×; catalog #LC2673, Thermo Fisher Scientific) and deionized water. Samples were loaded on NativePAGE 3–12%, Bis-Tris gels (catalog #BN1001BOX, Thermo Fisher Scientific). Gels were run using 1× NativePAGE Anode buffer and 1× NativePAGE Dark Blue Cathode buffer as per manufacturer’s instructions. Gels were fixed and de-stained as per manufacturer’s instructions and then imaged using a FluorChem E system (Protein Simple). Images were analyzed using the Gel Analyzer function of ImageJ (Schneider et al., 2012) and the molecular size markers run on either side of the recombinant proteins, allowing us to obtain the median size of the oligomers for each recombinant protein. The area under the curve is shown as a function of oligomer sizes from <480 to 1236 kDa, with the median shown for each recombinant protein.

### Solubility assays

As above, cells were transfected with 2.5 μg of pcDNA 3.1(+) plasmids expressing either WT, T148D, or T148A crystallins or an empty vector (EV) and were seeded in six-well plates. The next day, transfected cells were incubated either in DMEM-NG + 10% FBS or serum-free DMEM-NG for 4 or 24 h. Cells transfected with EV served as an experimental control. Following incubation, cells were harvested on ice in chilled RIPA buffer (100 mm Tris pH 7.5, 3 mm EGTA, 5 mm MgCl_2_, 0.5% Triton X-100, 1 mm PMSF, 1× complete EDTA-free protease inhibitor cocktail tablet; Roche Diagnostics) and subjected to immunoblot analyses.

### Protein uptake assay

Differentiated R28 cells were allowed to grow on laminin-coated plates for 36 h. Recombinant WT, T148D, or T148A crystallins were supplemented to growth media at a concentration of 500 ng/ml. Protein uptake in R28 cells was tested in DMEM-NG versus serum-free DMEM-NG, the presence and absence of BSA for 4 h. Protein uptake by R28 cells was also tested under stress by incubating cells in serum-free DMEM-NG (with 20 mm mannitol), DMEM-HG (DMEM-NG with 20 mm glucose), or DMEM-HG with 100 ng/ml TNFα. Following incubation, cells were harvested on ice in chilled RIPA buffer and subjected to immunoblot analyses.

### Proteinase K susceptibility assay

Differentiated R28 cells were allowed to grow on laminin-coated six-well plates for 36 h. Recombinant WT αA-crystallin was supplemented with growth media at a 500 ng/ml concentration for 2, 4, and 24 h. The cell lysates were then subjected to proteinase K treatment as adopted by Ruebsam et al., 2018. Briefly, cell lysates were treated with 100 ng proteinase K (Millipore Sigma) in a reaction containing 10 mm Tris-HCl (pH 7.4) with or without Triton X-100 (1%) for 30 min at 37°C. The reaction was stopped by the addition of loading buffer and heated at 70°C for 10 min. A control sample was treated the same way, aside from the omission of the enzymes. Protein levels were then assessed by immunoblotting as described below.

### Immunoblotting analyses

Protein concentrations were measured with the Pierce BCA reagent, and all samples and conditioned media were adjusted for equal protein concentration. To assess the purity of the recombinant protein preparations, 50 ng of pure protein was subjected to immunoblot analyses. For protein uptake experiments, cells were homogenized by sonication in RIPA buffer as described previously (Ruebsam et al., 2018) and 35 μg of the total cell lysate was loaded on 4–12% NuPage Bis-Tris gels (Thermo Fisher Scientific). Gels were run in MES buffer (Thermo Fisher Scientific) as per manufacturer’s instructions. Western blot transfer was conducted on nitrocellulose membranes using the Mini Trans-Blot cell (catalog #1703930, Bio-Rad) at 160 V for 1 h at 4°C. For solubility assays, RIPA-soluble protein lysates were collected, and insoluble pellets were resuspended after PBS wash by sonication in Urea buffer (10 mm Tris pH 7.5, 150 mm NaCl, 5 mm EGTA, 5 mm MgCl_2_, 1 mm DTT, 0.1% Triton X-100, 0.2 mm PMSF, 9 m urea). Soluble samples were adjusted for equal protein concentration, while insoluble samples were adjusted for equal volume. Samples were loaded on 4–12% NuPage Bis-Tris gels and run in MES buffer as per manufacturer’s instructions, at 110 V. Cell lysates and conditioned media were immunoblotted for αA-crystallin (sc-28306, Santa Cruz Biotechnology) and β-actin (MAB-1501, Millipore) as a loading control. Solubility was measured as a ratio of insoluble αA-crystallin to total αA-crystallin for each condition (using the Gel Analyzer function in ImageJ; Schneider et al., 2012), normalized to WT, and data were analyzed using the GraphPad Prism software module (GraphPad Software).

### Cell death assay

Cell death rates were assessed by DNA Fragmentation ELISA or TUNEL staining. For the DNA fragmentation ELISA (Roche Diagnostics), R28 cells were seeded in a 96-well plate at a density of 1 × 10^5^ cells per well and incubated with 100 μl of conditioned medium for 4 h before being processed according to the manufacturer’s instructions and as previously described (Ruebsam et al., 2018). The colorimetric signal was measured with a fluorescence plate reader in a FLUOstar OMEGA plate reader (BMG Labtech) with excitation at 405 and 490 nm.

For TUNEL staining, cells were seeded on glass coverslips as previously described. Following incubation, the coverslips were fixed in 4% PFA and stained for TUNEL (DeadEnd Fluorometric TUNEL System, Promega) according to manufacturer’s instructions. Briefly, the samples were incubated with fluorescent-labeled dUTP and TdT enzymes. The nuclei were visualized by Hoechst staining. Images were captured on a Leica DM6000 fluorescent microscope. Nuclei and TUNEL-positive cells were counted using ImageJ (Schneider et al., 2012), and data were analyzed using the GraphPad Prism software module (GraphPad Software).

For primary RGC, characterization of the primary cells was performed by immunostaining with RGC-specific markers, β3-tubulin (Biolegend, catalog #801201), Neurofilament-H (NF-H; Millipore, catalog #NE1023), and RNA-binding protein with multiple splicing (RBPMS; Rodriguez et al., 2014; Genetex, catalog #GTX118169). For cell survival experiments, the coverslips were first subjected to TUNEL staining as described above. After TUNEL, the coverslips were immunostained with RBPMS antibody and secondary Alexa Flour 594-labeled antibody (Invitrogen, A21207). All Immunostainings were visualized, and images were captured using Leica DM6000 fluorescent microscope. Cells staining positive for RBPMS and both TUNEL and RBPMS were counted using the Imaris software module (Bitplane AG). The data were analyzed using GraphPad Prism (GraphPad Software).

### Statistics

For immunoblot experiments, the data were normalized to the housekeeping signal as a control before analysis. ANOVA models with heterogeneous variances, adjusted for the replication of the experiment, were fit to the data to assess differences between test and control group. Analyses were performed using nonrepeated-measures ANOVA, followed by the Tukey’s *post hoc* tests for multiple comparisons, whereas two-tailed *t* test was used for a single comparison. A *p* value <0.05 was considered significant. All experiments were conducted following the Association for Research in Vision and Ophthalmology Resolution on the Care and Use of Laboratory Animals and approved by the Institutional Animal Care and Use Committee of the University of Michigan (protocol no. PRO-00009143, approved 7/9/2019).

## Results

### Expression and secretion of αA-crystallin in MGCs

MGCs are instrumental in maintaining neuronal homeostasis in the retina, with defined functions ranging from the recycling of neurotransmitters to controlling ionic and water equilibrium (Dulle and Fort, 2016). Our previous work emphasized the upregulation of αA-crystallin in the glia and ganglion cell layers of retinal tissue from human donors with diabetes compared with nondiabetic controls. Furthermore, growth media from rMC-1 cells overexpressing αA-crystallin efficiently promoted survival of R28 cells under serum starvation-induced apoptotic stress (Ruebsam et al., 2018). To further investigate the role of αA-crystallin in MGCs, we compared the relative expression of αA-crystallin in rMC-1 and primary MGCs isolated from AKO mice. Cells from AKO mice were used throughout this study to avoid potential confounding effect of endogenously expressed and induced WT αA-crystallin. Thus, cells lacking endogenous αA-crystallin expression were transfected with either EVs or vectors driving expression of the WT protein, the functionally enhanced phosphomimetic (T148D), or the nonphosphorylatable (T148A) analog, and αA-crystallin expression was verified by immunoblot.

**Figure 1.**
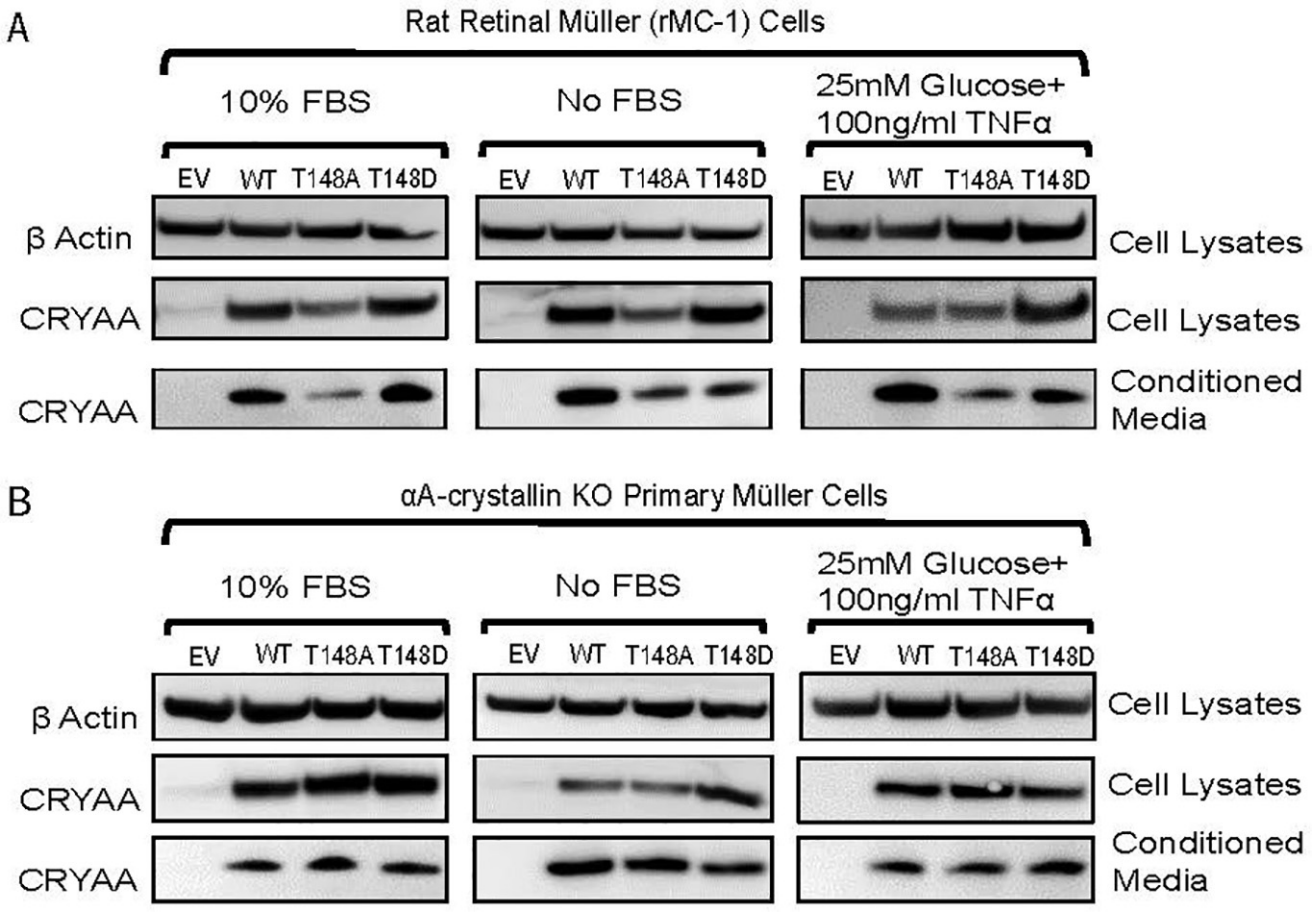
Expression and secretion of αA-crystallins by Müller cells. αA-crystallin (CRYAA) expression was observed in the cell lysates and the concentrated growth (conditioned) media. Rat retinal Müller (rMC-1) cells (***A***) and primary Müller cells (***B***) isolated from AKO mice were transfected with either EV, wild-type αA-crystallin (WT), αA-crystallin phosphomimetic (T148D), and the nonphosphorylatable form of αA-crystallin (T148A). Twenty-four hours after transfection, cells were either exposed to normal media (DMEM-NG + 10% FBS), serum starvation (DMEM-NG No FBS), or diabetic-like stress (DMEM-NG + 20 mm glucose +100 ng/ml TNFα) for 4 h.

As we previously reported, WT, 148A, and 148D αA-crystallin expressed well in transfected rMC-1. We also observed corresponding levels of secreted proteins in the cell culture media (conditioned media; Fig. 1*A*, left panel). Additionally, the expression of all three crystallin constructs was consistent in the cell lysate and conditioned media under normal conditions as well as under conditions of metabolic and “diabetes-like” stress (Fig. 1*A*, middle and right panel). Importantly, we also report that primary MGCs could be transfected with the same vectors, leading to expression levels and secretion comparable to those seen in rMC-1 (Fig. 1*B*). Similar to rMC-1, our data also clearly show that stress exposure does not affect the expression and secretion of any of our αA-crystallin constructs.

### Neuroprotective potential of MGC-secreted αA-crystallin

**Figure 2.**
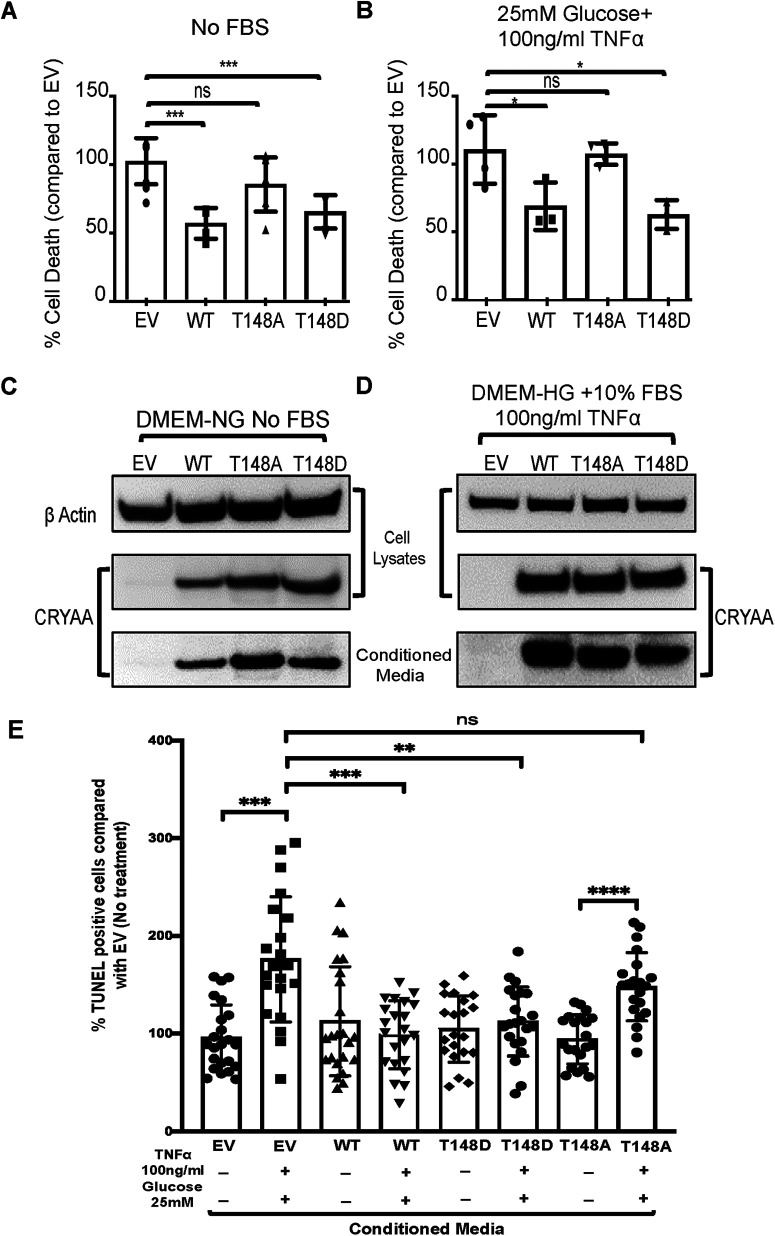
MGC-secreted αA-crystallin promotes neuronal cell survival under stress. The relative viability of rat retinal neuronal (R28) cells under (***A***) serum starvation stress and (***B***) “diabetes-like” condition, following supplementation of “conditioned” media from MGCs overexpressing αA-crystallin; *p ≤ 0.05, **p ≤ 0.01, ***p ≤ 0.001, significantly different from respective EV-transfected cells. Representative endpoint statistics result of DNA fragmentation ELISA from three replicates, with relative significance determined by one-way ANOVA followed by the Tukey’s *post hoc* tests. Immunoblotting analyses reveal a similar expression pattern of WT, T148A, and T148D crystallins in comparison to EV control under (***C***) serum starvation stress and (***D***) “diabetes-like” condition. ***E***, The relative viability of primary, AKO mouse RGCs under basal and stress conditions following supplementation of “conditioned media” (CM) from MGCs overexpressing αA-crystallin. Representative endpoint statistics result of TUNEL from three to four fields from three coverslips per condition of three replicates, with relative significance determined by one-way ANOVA followed by Tukey’s *post hoc* tests. The data were expressed as mean ± SD and statistically significant differences are reported; **p ≤ 0.01, ***p ≤ 0.001, ****p ≤ 0.0001, significantly different from respective EV-transfected cells.

Overexpression of αA-crystallin in multiple cell models has demonstrated its anti-apoptotic potential under conditions of stress-induced cell death (Liu et al., 2004; Pasupuleti et al., 2010; Losiewicz and Fort, 2011; Christopher et al., 2014; Ruebsam et al., 2018). To investigate the protective potential of MGC-secreted αA-crystallin, we tested the effect of conditioned media obtained from αA-crystallin transfected primary MGCs on retinal neurons subjected to metabolic stress. Supplementation of conditioned media from MGCs overexpressing WT and T148D crystallin highly promoted R28 cell survival under serum starvation-induced metabolic stress, as evidenced by the reduction in cell death by 45% and 37%, respectively. Similarly, in “diabetes-like” stress, conditioned media from MGCs overexpressing WT or 148D αA-crystallin resulted in 38% and 44% reduction in R28 cell death, respectively. Supportive of the key role of T148 phosphorylation, media from T148A overexpressing MGCs was ineffective in promoting R-28 cell survival in either stress (Fig. 2*A*,*B*). Immunoblot analysis of the cell lysate and conditioned media confirmed that this difference in protective effect was not because of lower levels of expression or secretion of T148A (Fig. 2*C*,*D*). We then tested the effect of conditioned media on primary, AKO mouse RGCs. As in R28 cells, supplementation of conditioned media from MGCs overexpressing WT and T148D crystallin highly promoted RGC survival under “diabetes-like” stress, while media from T148A overexpressing MGCs did not (Fig. 2*E*). Taken together, these data clearly demonstrate the neuroprotective potential of αA-crystallin and validate a paracrine role of MGC-secreted αA-crystallin in promoting neuronal cell survival under stress.

### Characterization of recombinant αA-crystallins

**Figure 3.**
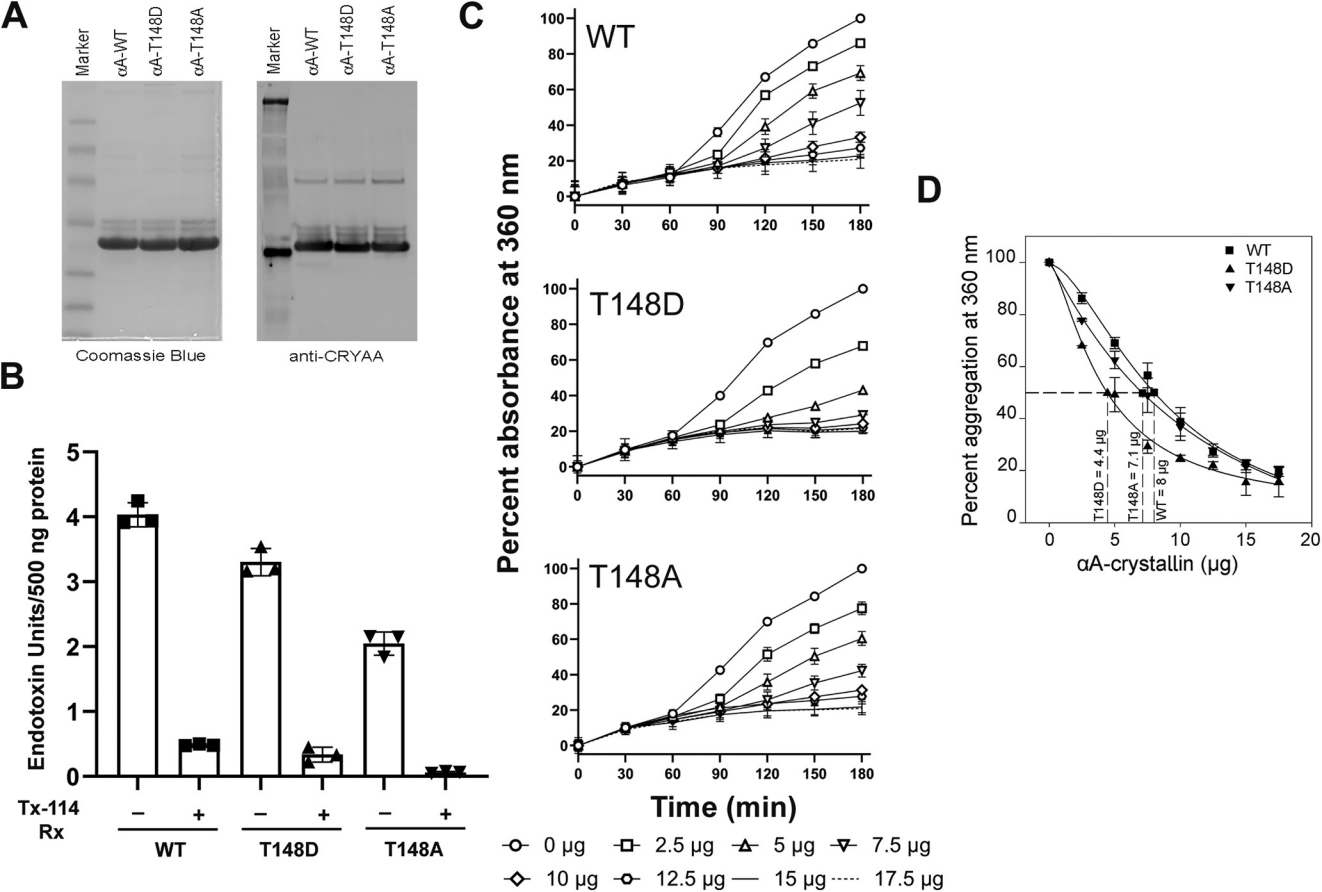
Characterization of recombinant αA-crystallins. ***A***, BL21 purified αA-crystallins were analyzed for purity using SDS-PAGE (top panel) and Western blotting (bottom panel), respectively. ***B***, Triton X-114 treatment of purified αA-crystallins drastically reduces their relative endotoxin content in comparison to nontreated controls; 500 ng of each of the purified proteins was subjected to endotoxin estimation using the LAL assay kit. ***C***, Chaperone assays with ADH show a αA-crystallin concentration-dependent decrease in ADH aggregation, monitored as relative absorbance at 360 nm. The range of αA-crystallin concentrations used is depicted in the legend**. *D***, *In vitro* chaperone activity assays reveal an enhanced chaperone function of T148D crystallin over αA-WT (*n* = 3). The data are represented as mean ± SD and statistically significant differences are reported; **p ≤ 0.01, ***p ≤ 0.001, ****p ≤ 0.0001, significantly different from respective EV-transfected cells.

Our experiments with secreted αA-crystallin highlighted the neuroprotective potential of extracellular WT and T148D crystallins in promoting neuronal cell survival exposed to serum starvation and “diabetes-like” conditions. This prompted us to assess whether our observation from the conditioned media experiments could be recapitulated using purified, recombinant αA-crystallin proteins. All three proteins, WT, T148A, and T148D were scaled up from BL21(DE3) pLysS cells expressing the specified constructs and purified by size exclusion chromatography. As shown in Figure 3*A*, the three purified proteins show a high degree of purity, as validated by SDS-PAGE and immunoblotting analyses. Because recombinant proteins purified from bacterial sources are often contaminated with bacterial endotoxins, which compromises their use *in vivo*, our protein preparations were treated with Triton X-114, a treatment routinely used to promote efficient endotoxin removal (Teodorowicz et al., 2017). Qualitative analysis of the recombinant protein preparations post-Triton X-114-mediated phase separation confirmed the >90% reduction in the total endotoxin content (Fig. 3*B*).

αA-crystallins were initially characterized in the eye lens as chaperone proteins, efficiently preventing nonspecific protein aggregation and promoting organ transparency. *In vitro*, we tested the relative chaperone function of WT, T148D, and T148A crystallins by employing aggregation kinetics assays. As previously shown, EDTA-induced aggregation of ADH was suppressed by αA-crystallins in a concentration-dependent manner (Fig. 3*C*). Consistent with enhancing αA-crystallin chaperone activity by its phosphorylation on T148, the T148D mutant was significantly more effective at preventing ADH aggregation *in vitro* (Fig. 3*D*). While the WT αA-crystallin exhibited an IC_50_ of 8 μg, that of the T148D crystallin mutant was 4.4 μg, demonstrating an increase in chaperone efficacy of 45%. The phosphorylation of αA-crystallin on T148, therefore, results in an enhancement of its chaperone function.

### Stress-induced insolubility of αA-crystallin

**Figure 4.**
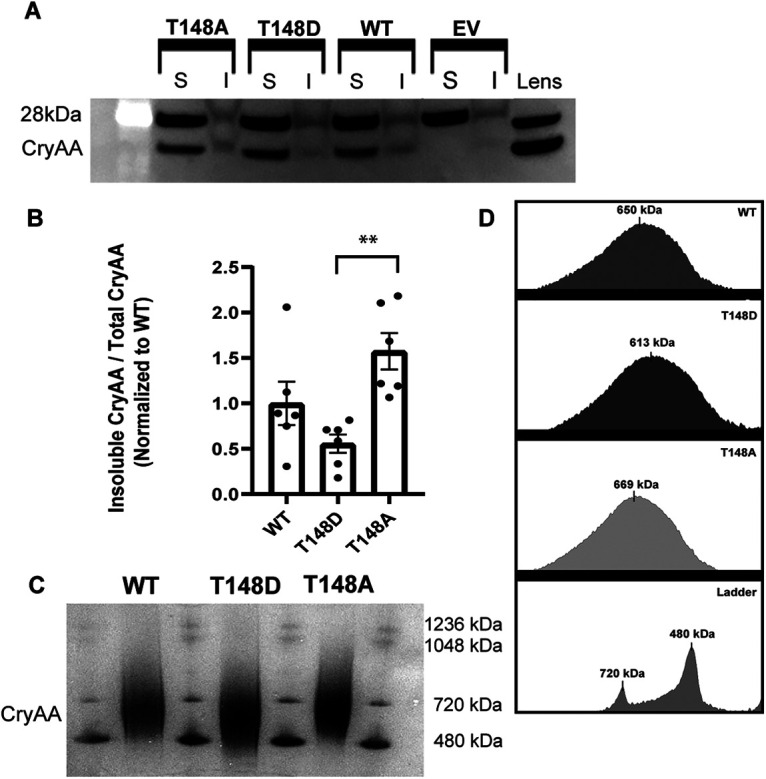
Phosphomimetic and nonphosphorylatable mutants of αA-crystallin exhibit differences in stress-induced solubility and oligomeric profile. ***A***, Representative blot showing relative amounts of soluble (S) and insoluble (I) αA-crystallin after 24 h of serum deprivation. ***B***, Solubility differences are expressed as a ratio of insoluble αA-crystallin to total αA-crystallin for each condition, normalized to WT. Data are represented as mean ± SD. Statistical comparisons between groups were calculated by one-way ANOVA followed by Tukey’s *post hoc* tests (**p < 0.01). ***C***, Representative Native gel showing the oligomeric profiles of WT, T148D, and T148A αA-crystallin. ***D***, Graphical representation of oligomeric profiles of the native gels (representative of 3 independent experiments). Median oligomer size for each recombinant protein is shown, rounded to the nearest kilodalton.

WT, T148D, and T148A αA-crystallin expressed in R28 cells exhibited no differences in basal solubility (Fig. 4*A*). However, following 4 h of serum starvation, T148A trended toward higher insolubility, and T148D trended toward lower insolubility (data not shown), an effect confirmed and enhanced after 24 h of serum starvation (Fig. 4*B*). This observation indicates that phosphorylation on residue T148 plays a key role in promoting αA-crystallin’s function, including by reducing stress-induced insolubility.

### T148 phosphorylation-dependent changes in oligomer size

αA-crystallin, along with its close relative αB-crystallin is known to exist as larger oligomers, we thus assessed how this phosphorylation impacts the oligomeric state of αA-crystallin. T148A αA-crystallin formed slightly larger oligomers (median 669 kDa) than the WT αA-crystallin (median 650 kDa), whereas T148D formed substantially smaller oligomers (median 613 kDa; Fig. 4*C*,*D*). These data are clearly supportive of the T148 phosphorylation state impacting oligomeric and potentially aggregate formation. This could also partially explain the solubility data as the decreased oligomeric size observed for the T148D mutant could promote the protein’s solubility under stress conditions. Together, the solubility and oligomeric data are consistent with the relative prosurvival potential of the mutants. As αA-crystallin becomes more insoluble and/or forms larger oligomers, less is likely available to serve normal chaperone and protective roles.

### Uptake of recombinant αA-crystallins

**Figure 5.**
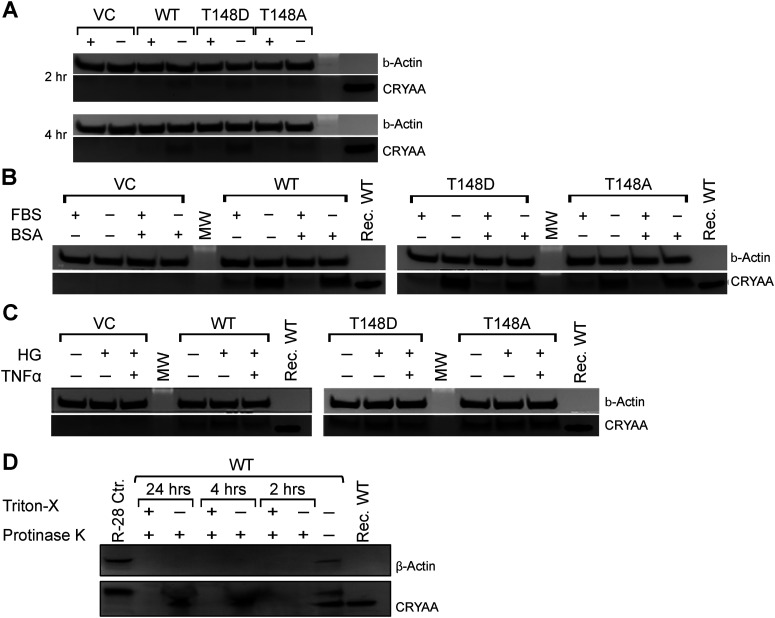
Selective uptake of recombinant αA-crystallins by R28 cells. All recombinant proteins were supplemented at a concentration of 500 ng/ml. ***A***, Time-dependent uptake of recombinant αA-crystallins by R28 cells under serum starvation induced metabolic stress. Uptake of αA-crystallins in R28 cells was dependent on the presence of serum (***B***) and specificity of induced “diabetes-like” conditions (***C***) as mimicked by supplementation of DMEM-NG ± 10% FBS ± 1% BSA and DMEM-HG (25 mm) + 10% FBS ± 100 ng/ml TNFα, respectively. ***D***, Supplemented recombinant αA-crystallins were internalized in the R28 cells as assessed by Proteinase K susceptibility assay.

Our previous study showed that conditioned media from MGCs expressing αA-crystallins WT and T148D greatly reduced stress-induced R28 cell death. Before testing the neuroprotective efficacy of the different recombinant αA-crystallins, we first characterized the specificity of their uptake in R28 cells. As expected, supplementation of recombinant αA-crystallins to differentiated R28 cells showed a gradual increase in their uptake as a function of time (Fig. 5*A*). We then assessed the impact of stress on protein uptake and showed that serum starvation was associated with an increased uptake of all recombinant proteins, including T148A, although slightly less than WT and T148D αA-crystallins. Since T148A is taken up by the cells under stress, it can be asserted that the level of protein uptake is not solely responsible for the relative protective efficacy of the different recombinant proteins.

To eliminate the possibility that the increased uptake of recombinant proteins observed in serum starvation is facilitated by the lack of interactions that would otherwise occur with components of FBS, we spiked the growth media with saturating concentrations of BSA (1%). Supplementation of BSA did not impact protein uptake, suggesting the difference in uptake of proteins as part of the stress response (Fig. 5*B*). The level of protein uptake was further investigated by characterizing protein uptake under “diabetes-like” conditions (Fig. 5*C*). Protein uptake progressively increased in cells exposed to “diabetes-like” conditions (HG, HG+TNFα lanes) and is independent of T148 mutation (Fig. 5*C*). Collectively, our data demonstrate that T148 mutation does not dramatically impact its uptake by R28 cells in a way that could significantly affect its observed neuroprotective efficacy under stress.

Following the uptake assay of recombinant proteins, the R28 cells were further assessed for the internalization of these proteins. The obtained results have demonstrated the time-dependent marked expression of recombinant WT αA-crystallin in R-28 cells in the intact cell membrane during protease digestion. On the contrary, the intracellular access of protease in R-28 cells led to the complete digestion of WT αA-crystallin, confirming the internalization of αA-crystallin recombinant proteins in cells on its extracellular supplementation (Fig. 5*D*).

### Effect of αA-crystallin supplementation on neuronal cell viability

**Figure 6.**
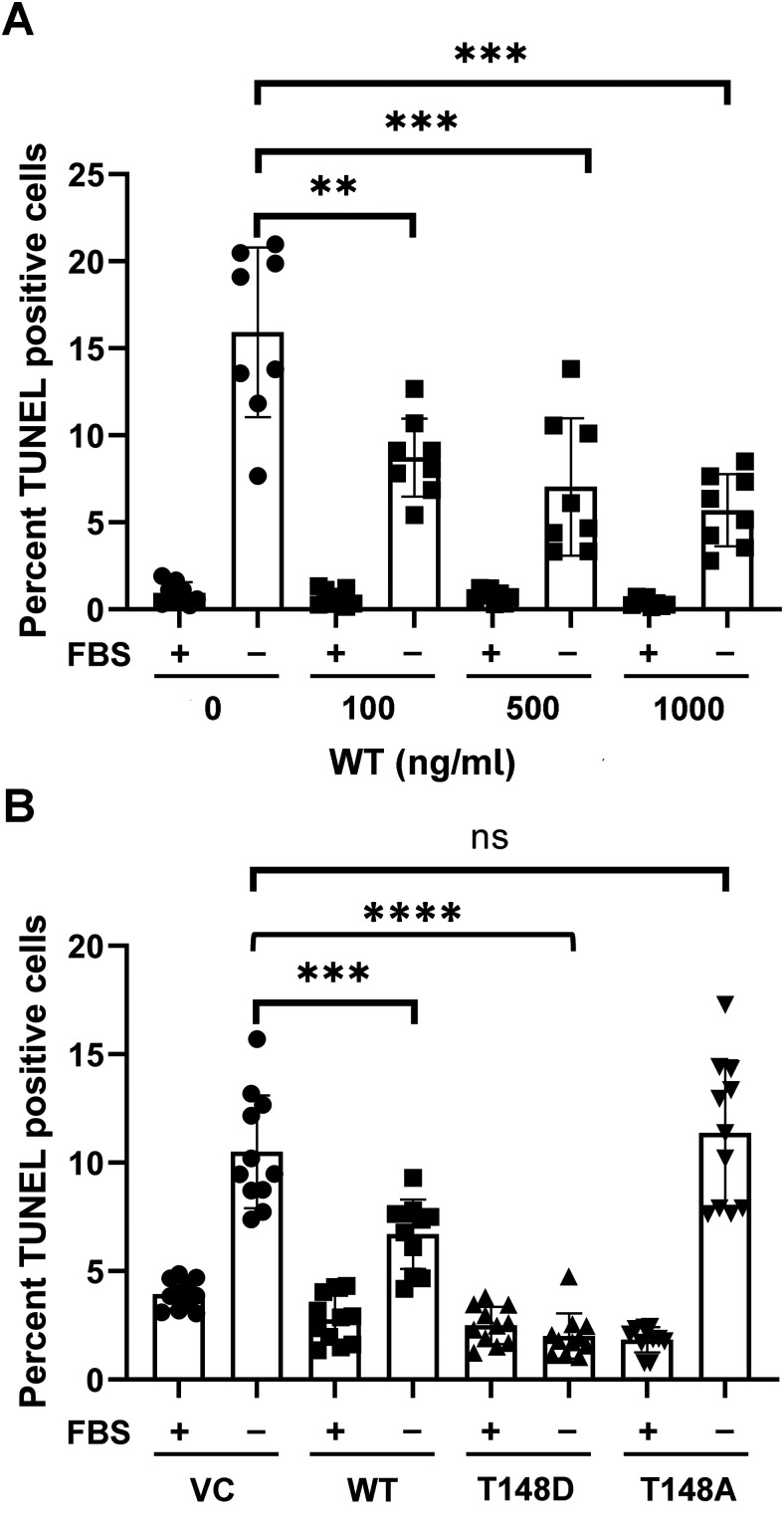
Effect of αA-crystallin supplementation on R28 cell viability under stress. All proteins were supplemented to R28 cells in DMEM-NG ± 10% FBS. Following treatment, cell viability was assessed by TUNEL staining. ***A***, Supplementation of WT suppresses serum starvation induced R28 cell death in a dose-dependent manner. ***B***, T148D crystallin supplementation efficiently prevents R28 cell death under serum starvation induced metabolic stress compared with WT and T148A. Data are representative of four fields from three coverslips per condition and are represented as mean ± SD from ***p ≤ 0.0005, ****p ≤ 0.000005, significantly different from respective EV.

To test the effect of uptake of recombinant αA-crystallins on cell viability under conditions of stress, we sought to establish a dose–response effect of αA-crystallin concentration on R28 cell viability. External supplementation of WT αA-crystallin efficiently prevented serum starvation-induced R28 cell death in a dose-dependent manner (Fig. 6*A*) as validated by TUNEL staining. Approximately 60% reduction in R28 cell death was observed following incubation with 500 ng/ml WT αA-crystallin, and this dose was selected to assess the relative neuroprotective efficacy of T148D and T148A crystallins in comparison to WT. Figure 5*B* summarizes the relative efficacies of 500 ng/ml WT, T148D, and T148A crystallins in promoting R28 cell survival in response to serum starvation-induced apoptotic stress. Compared with control, incubation with 500 ng/ml T148D crystallin resulted in ∼85% increased cell viability in comparison to WT (∼30%). Incubation with 500 ng/ml T148A did not promote R28 cell viability, further validating the key role of phosphorylation of αA-crystallin on T148 for its neuroprotective function (Fig. 6*B*).

**Figure 7.**
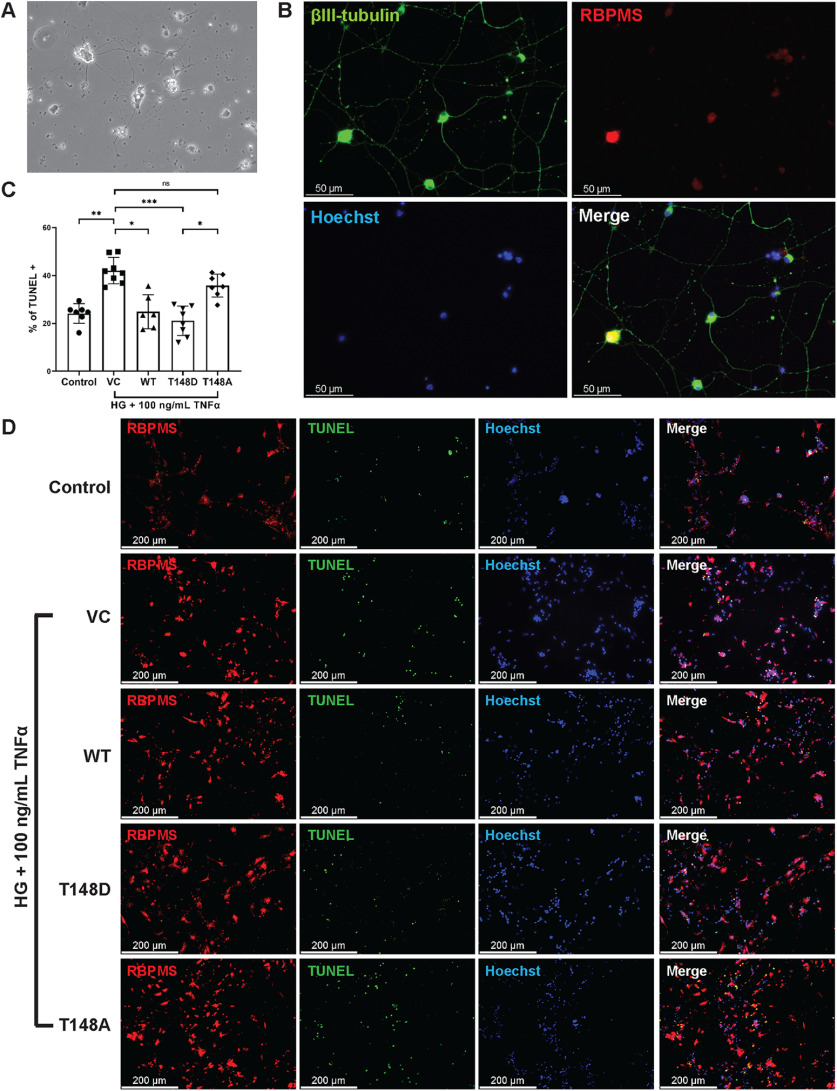
Exogenous αA-crystallin protects primary mice retinal ganglion cells under stress. (***A***) Seven days post seeding, the RGCs show prominent neural processes. (***B***) Immunofluorescence analyses highlight prominent staining for neuron-specific βIII-tubulin (green), RBPMS (red) and Hoechst (blue). (***C***) Statistical analyses of RGC viability following exposure to stress. Percentage of apoptotic cells (TUNEL positive) in all RGCs (RBPMS positive) were analyzed. Data are represented as mean ± S.D. Statistical comparisons between groups were calculated by One-Way ANOVA followed by Tukey post-hoc tests. (**p ≤ 0.01), (***p ≤ 0.001), (****p ≤ 0.0001), significantly different from respective EV-transfected cells. (***D***) Vehicle control (VC), Recombinant wild type αA-crystallin (WT), αA-crystallin phosphomimetic (T148D), and the non-phosphorylatable form of αA-crystallin (T148A) were supplemented to RGCs at a 500 ng/ml concentration and incubated for 8 hours with 25 mm D-glucose (HG) and 100 ng/ml TNFα for 8 hours. Cells incubated with 5 mm glucose (NG) served as an experimental control. RGC survival under stress was assessed by TUNEL staining (green), and cells were later stained for RBPMS (red).

To confirm the neuroprotective efficacy of recombinant αA-crystallins in promoting neuronal cell survival, we further tested the ability of the recombinant protein supplementation on the survival of primary RGCs under “diabetes-like” conditions. As to avoid potential complications because of induction of endogenous αA-crystallin, RGCs were also isolated from the retinas of AKO mice, and the purity of the RGC preparation was assessed by staining for neuronal cell-specific markers (Fig. 7*A–C*). RGCs cell death under “diabetes-like” conditions was then analyzed by TUNEL and RBPMS co-staining. Consistent with the effect seen in differentiated R28 cells, supplementation with 500 ng/ml of WT or T148D αA-crystallins were highly protective of RGC cells exposed to metabolic stress (Fig. 7*D*,*E*). Also similar to what was observed in R28 cells, co-incubation with T148D was slightly more protective than WT while T148A crystallin completely failed to prevent cell death, emphasizing an inherent role of T148 phosphorylation on the neuroprotective efficacy of αA-crystallin.

## Discussion

Our present study has shown that primary MGCs can secrete αA-crystallin and that secreted αA-crystallin presents with significant neuroprotective abilities for retinal neuronal cells exposed to metabolic stresses. Supportive of a paracrine function of the secreted protein and therapeutic potential for αA-crystallin recombinant proteins was the demonstration of its increased uptake in stressed retinal neurons. Furthermore, analysis of the biochemical and biophysical properties of these recombinant proteins revealed an increased chaperone activity, smaller oligomer assembly, and an increased solubility of the T148D αA-phosphomimetic, consistent with its enhanced protective effect. Overall, our study shows that supplemented αA-crystallin recombinant proteins are neuroprotective for primary retinal neurons exposed to metabolic stress and that αA-crystallin T148D phosphomimetic mutant presents with enhanced therapeutic ability.

Müller glia has been shown to release trophic factors which regulate the various aspects of retinal neuronal circuitry during the process of synaptogenesis, differentiation, neuroprotection, and survival of photoreceptors and RGCs in the retina (de Melo Reis et al., 2008). MGCs, astrocytes, and microglia also play an important role in the metabolism, the phagocytosis of neuronal debris, the release of certain neurotransmitters, and the release of trophic factors apart from providing structural support (Vecino et al., 2016). They are also reported to be involved in the inflammation associated with the pathophysiology of diabetic retinopathy (DR), with special emphasis on the functional relationships between glial cells and neurons (Rübsam et al., 2018). MGCs are an important source of numerous prosurvival factors under inflammatory conditions to exert neuroprotection, a potentially key point in patients with DR since they have higher levels of both inflammatory cytokines and neurotransmitters in their vitreous (Boss et al., 2017).

More recently, it also has been observed that nontoxin-induced Müller cell ablation is detrimental for neurons further supporting their necessity for neuronal viability (Fu et al., 2015). Stem cell-derived RGC-like cells survival was substantially enhanced when co-cultured with adult Müller cells or supplemented with Müller cell-conditioned media and significantly increased their neurite length (Pereiro et al., 2020). Confluent retinal MGC substrates and its conditioned medium were also reported to significantly increase the survival of cultured porcine RGCs (Garcia et al., 2002). Our current study has also demonstrated that retinal MGCs were able to secrete αA-crystallin, and incubation of either R28 retinal neuronal cells or primary AKO mouse RGCs with the conditioned media resulted in a significant decrease in the cell death induced by metabolic stress. Our study further confirmed the importance of T148 phosphorylation in the neuroprotective function of αA-crystallin as evidenced by the greater protection of retinal neurons by the phosphomimetic mutant conditioned media, while the nonphosphorylatable mutant conditioned media had no effect.

The effect of phosphorylation on the structure and function of α-crystallin has largely been studied for αB-crystallin, owing to its ubiquitous distribution and upregulation under stress and disease conditions. Studies investigating chaperone and anti-apoptotic activity of phosphorylated αB-crystallin mostly support a prochaperone and anti-apoptotic enhancer role of this phosphorylation under various cellular stresses while underlying a more complex function during development and cancer (Morrison et al., 2003; Jeong et al., 2012; Lee et al., 2016). In the meantime, the effect of phosphorylation on the chaperone function and the anti-apoptotic activity of αA-crystallin have evaded diligent investigation.

Takemoto first reported an increase in the phosphorylation of αA-crystallin on S122 from donor lens tissue in an age-dependent fashion (Takemoto, 1996). 2D gel electrophoresis on lens tissue of 14-week C57BL/6 mice identified T148 in addition to S122 as sites of phosphorylation on αA-crystallin (Reddy et al., 2006). A recent study from our lab was the first to identify T148 phosphorylation *in vivo* from retinal tissue samples from human donors (Ruebsam et al., 2018). The modification was dramatically reduced in donor samples with diabetes, suggesting an inherent role for T148 phosphorylation of αA-crystallin in the pathophysiology of DR. Overexpression of the αA-crystallin phosphomimetic T148D conferred protection to R28 neuronal cells to serum starvation-induced apoptosis over its nonphosphorylatable analog T148A. The current study, therefore, investigated the structural and functional consequences of T148 phosphorylation on αA-crystallin.

Mutations in αA-crystallin have been shown to influence its chaperone activity. Recombinant αA-T148D crystallin exhibited maximal efficiency in preventing EDTA-induced aggregation of ADH over WT and T148A crystallin. In conjunction with the observed cytoprotective effect observed in the earlier study, it shows that phosphorylation of T148 enhances the chaperone function and the associated anti-apoptotic function of retinal αA-crystallin. Both α-crystallin proteins have been shown to associate into large oligomeric structures with molar masses ranging from 400 to 700 kDa. Our study has shown that phosphorylation of αA-crystallin was directly influencing the oligomeric assembly of αA-crystallin *in vitro*. Native gel analysis of recombinant αA-crystallins suggests a change in the polydispersity profile of T148D crystallin, which showed an increased predisposition to form smaller oligomeric assemblies when compared with the WT and T148A αA-crystallin. Since the chaperone activity of α-crystallin has been shown to be modulated by hydrophobic “patches” distributed along with its monomeric structure (Rao et al., 1998; Datta and Rao, 1999). The observed oligomeric shift in our present study could translate into a higher number of smaller oligomers exerting their chaperone action. Studies have also demonstrated that exposure of hydrophobic residues by structural modification facilitates chaperoning in α-crystallin proteins whereas the flexible carboxy-terminal extension also contributes to the chaperone activity by enhancing the solubility (Ruebsam et al., 2018; MacRae, 2000). The change in oligomeric profile was less pronounced for T148A crystallin, which was to be expected, as the recombinant WT crystallin protein used in this experiment was similarly unphosphorylated. However, this difference may become more pronounced in the cellular environment as WT αA-crystallin becomes phosphorylated and explains the lack of protective ability of T148A recombinant proteins *in vitro*.

αA-crystallin was originally described as an endogenous neuroprotective factor in retinal neurons, as exhibited in overexpression-based studies in hypoxic stress, or glaucomatous and other optic neuropathies (MacRae, 2000). Several studies have also demonstrated that αA-crystallin enhanced endogenous expression has potential as the therapeutic strategy to protect and rescue neurons from degeneration associated with metabolic or hypoxic stress (MacRae, 2000; Ying et al., 2014). Similarly, exogenous supplementation of αA-crystallin via intravitreal injections was associated with significantly decreased levels of GFAP in both the retina and the crush site following the third day of optic nerve crush injury and induced astrocytes architecture remodeling at the crush site (Piri et al., 2016). In the increased intraocular pressure model, intravitreal injection of αB-crystallin was also able to increase RGCs survival and function, as measured by functional photopic electroretinogram, retinal nerve fiber layer thickness, and RGC counts (Shao et al., 2016). Another study has reported the enhanced rate of survival in the axotomized axons beyond the crush site after a single intravitreal administration of α-crystallin at the time of axotomy (Anders et al., 2017). Together with these previous reports, the present study strongly supports the protective potential of functionally enhanced αA-crystallin recombinant proteins against neurodegeneration.

In conclusion, our study demonstrates for the first time that the exogenous supplementation of αA-crystallin, especially its functionally enhanced mutant, promotes retinal cell survival under metabolic stress. Altogether, our data show that αA-crystallin recombinant proteins present a strong potential to reduce neuronal cell death during acute stresses and that its T148D phosphomimetic mutant form could be an interesting option in chronic diseases such as diabetes, because of its improved biochemical properties and enhanced functionality. *In vivo* studies, including in diabetes models are now essential to further demonstrate the potential of this approach and validate the neuroprotective effect of functionally enhanced αA-crystallin recombinant proteins. These studies will also be key in characterizing the mechanisms of action of αA-crystallin *in vivo* to unveil αA-crystallin-specific involvement in the regulation of neurosurvival and neuroinflammation.

## Synthesis

Reviewing Editor: Lindsay De Biase, University of California Los Angeles Decisions are customarily a result of the Reviewing Editor and the peer reviewers coming together and discussing their recommendations until a consensus is reached. When revisions are invited, a fact-based synthesis statement explaining their decision and outlining what is needed to prepare a revision will be listed below. The following reviewer(s) agreed to reveal their identity: NONE. Note: If this manuscript was transferred from JNeurosci and a decision was made to accept the manuscript without peer review, a brief statement to this effect will instead be what is listed below.

The authors have addressed reviewer concerns regarding more detail in the figure legends as well as methods. Both reviewers agree that the manuscript is clearly written and data generally of high quality, although the findings are not necessarily surprising based on previous findings in the literature.

We ask that the authors shorten the discussion by synthesizing findings of key studies rather than listing detailed findings of individual studies. In the resulting space, we ask that the authors discuss what are the key open questions that remain to be addressed and critical next directions for this line of research. For example, studies in mouse models of diabetes or retinal explant organotypic slices cultures to further explore the roles of alpha-Acrystallin in the more complex multi-cellular setting of the intact retina.
