# Erratum: Nath et al., “Evidence for Paracrine Protective Role of Exogenous αA-Crystallin in Retinal Ganglion Cells”

**DOI:** 10.1523/ENEURO.0298-22.2022

**Published:** 2022-08-26

In the article “Evidence for Paracrine Protective Role of Exogenous αA-Crystallin in Retinal Ganglion Cells,” by Madhu Nath, Zachary B. Sluzala, Ashutosh S. Phadte, Yang Shan, Angela M. Myers, and Patrice E. Fort, which published online on February 15, 2022, an incorrect concentration was given for the cAMP used with the cell lines in the experiments. In the Materials and Methods subsection Cell lines, the final concentration noted in the first paragraph should appear as 250 μm. This error does not affect the conclusions of the article, and the online version has been corrected.

